# Future healthy life expectancy among older adults in the US: a forecast based on cohort smoking and obesity history

**DOI:** 10.1186/s12963-016-0092-2

**Published:** 2016-07-12

**Authors:** Bochen Cao

**Affiliations:** Population Studies Center, University of Pennsylvania, McNeil Building, 3718 Locust Walk, Philadelphia, PA 19104 USA

**Keywords:** Healthy life expectancy, Forecast, Mortality, Morbidity, Smoking, Obesity, Multi-state life table, Lee-Carter model

## Abstract

**Background:**

In the past three decades, the elderly population in the United States experienced increase in life expectancy (LE) and disability-free life expectancy (LE^ND^), but decrease in life expectancy with disability (LE^D^). Smoking and obesity are two major risk factors that had negative impacts on these trends. While smoking prevalence continues to decline in recent decades, obesity prevalence has been growing and is currently at a high level. This study aims to forecast the healthy life expectancy for older adults aged 55 to 85 in the US from 2011 to 2040, in relation to their smoking and obesity history.

**Methods:**

First, population-level mortality data from the Human Mortality Database (HMD) and individual-level disability data from the US National Health Interview Survey (NHIS) were used to estimate the transition rates between different health states from 1982 to 2010, using a multi-state life table (MSLT) model. Second, the estimated transition rates were fitted and projected up to 2040, using a modified Lee-Carter model that incorporates cohort smoking and obesity history from NHIS.

**Results:**

Mortality and morbidity for both sexes will continue to decline in the next decades. Relative to 2010, men are expected to have 3.2 years gain in LE^ND^ and 0.8 years loss in LE^D^. For women, there will be 1.8 years gain in LE^ND^ and 0.8 years loss in LE^D^. By 2040, men and women are expected to spend respectively 80 % and 75 % of their remaining life expectancy between 55 and 85 disability-free.

**Conclusions:**

Smoking and obesity have independent negative impacts on both the survival and disability of the US older population in the coming decades, and are responsible for the present and future gender disparity in mortality and morbidity. Overall, the US older population is expected to enjoy sustained health improvements and compression of disability, largely due to decline in smoking.

**Electronic supplementary material:**

The online version of this article (doi:10.1186/s12963-016-0092-2) contains supplementary material, which is available to authorized users.

## Background

Life expectancy (LE) in the US has climbed gradually over the past decades, reaching historic highs of 76.2 years for men and 81.0 years for women in 2010 [1]. Despite its utility as a summary indicator of mortality, life expectancy alone is not sufficient to measure the quality of population health. Whether the fall in mortality is accompanied by a fall in morbidity is also of great interest in health studies. Healthy life expectancy is hence often calculated using combined mortality and morbidity information, to summarize the changes in the quality of population health [2]. The most common forms used for measuring healthy life expectancy are disability-free life expectancy (LE^ND^) and life expectancy with disability (LE^D^), respectively defined as the average number of years one is expected to live without and with disability. In addition, the proportion of years living without disability (LE^ND^/LE) can be used as a relative measure for morbidity.

In order to better understand the quality of health among older adults in the US, one needs to study the mortality and morbidity of the population as well as the underlying epidemiological transitions that drive them. Two prominent factors that have shaped the current mortality and morbidity in the US are smoking and obesity. Particularly, the decline in smoking prevalence is largely responsible for the mortality fall in recent decades. Nearly 800,000 lung cancer deaths in the US were prevented due to the decline in smoking between 1975 and 2000 [3]. However, obesity (particularly Class II/III obesity) is thought to be responsible for an increasing proportion of deaths, as its prevalence has been growing in the past decades and remained high in recent years [4–9]. Additionally, smokers have higher chances of suffering from many chronic diseases (particularly lung cancer, cardiovascular diseases, and diabetes) [10–14], potentially leading to both higher rates and longer duration of disability among older adults [15–19]. Similarly, obesity is associated with many conditions that are disabling but not fatal, including diabetes, heart diseases, respiratory problems, arthritis, back pain, and other musculoskeletal conditions that limit mobility and daily activities [20–23].

Furthermore, the cohort patterns of the impact of smoking and obesity on health have been documented by many existing studies [5, 24, 25]. Hence, this is valuable information that can be applied to forecasting future health outcome of the population. The prevalence of obesity is projected to remain high, while the prevalence of smoking is expected to keep falling in the US over the next few decades [9, 26–28]. Although the population is smoking less, there is no consensus on whether this change is leading to fewer years spent with disability. Some studies claim that smoking is associated with both smaller LE^ND^ and smaller LE^D^, leaving never-smokers the same or even more years with disability [11, 29–32]. In contrast, others argue that smokers are subject to expansion of disability in both absolute and relative terms, despite their already relatively shorter life [33–35]. The mortality risks of obesity-related chronic diseases, such as cardiovascular diseases, strokes, and diabetes, have dropped over the last two decades owing to effective medical intervention and prevention [12, 36, 37]. This further extends life spent with disability for the obese individuals. Many studies accordingly conclude that obesity may have stronger impact on disability than mortality and creates extra burden for health care [14, 16, 29, 38].

Given that smoking and obesity affect mortality and morbidity differently, their trends combined have important implications for population health in the future. Although abundant studies have forecasted future mortality, few have attempted to do so for future morbidity. To date, there have been only few studies that forecast healthy life expectancy, among which only one is an application to the US population, and none of them account for the underlying factors that drive mortality and morbidity [39, 40]. As Wang and Preston [41] show, including a smoking covariate substantially reduces the anomalies in the shape and sex differences for parameter estimates which may otherwise be severely distorted as the projection period extends further. Additionally, King and Soneji [42] demonstrate that by incorporating smoking and obesity history in the US population, more informed and plausible mortality forecasts can be produced.

This study forecasts both LE^ND^ and LE^D^ from 2011 to 2040 for the US population between ages 55 and 85 in associations with its observed history of health behaviors at younger ages. A multi-state life table (MSLT) approach proposed by Majer et al. [40] is applied to estimate the transition rates among different health status. A modified Lee-Carter model that incorporates cohort smoking and obesity history will then be used to fit and forecast the obtained transition rates, based on which LE^ND^ and LE^D^ will be calculated.

## Data

Age- and gender-specific mortality rates are drawn from the Human Mortality Database (HMD) for the US population aged 55 to 85 for the observation period (1982–2010). The information for disability, smoking, and obesity is obtained from the Integrated Health Interview Series (IHIS), which maintains a harmonized set of public use data and documentation of the US National Health Interview Survey (NHIS) [43]. NHIS is a nationally representative cross-sectional survey of US non-institutionalized civilians, and is conducted annually by the National Center for Health Statistics. It collects comprehensive information about demographic, socioeconomic status, general health, health-related behaviors, and activity limitations. The sample used in this study contains observations of those that are 55 to 85 years old in the survey years 1982 to 2010.

The disability variable is constructed using questions that ask individuals’ limitations in activities due to chronic conditions. For surveys from 1982 to 1996, four categories are available, including: *not able to perform major activities*, *limited in amount/kind of major activities*, *limited in other activities,* and *not limited*. However, there are only three categories for surveys after 1996, including: *limited in any way*, *not limited in any way,* and *unknown*. In order to make the disability status comparable across surveys, an individual is considered to be disabled if he/she reports any limitations of activities at all. These limitations, due to physical, mental, or emotional problems, include: limitations with activities of daily living (ADL) that require help from others for personal care needs (e.g., walking, eating, bathing, dressing, or getting around inside the home); and limitations with instrumental activities of daily living (IADL) that require help from others in handling routine needs (e.g., everyday household chores, doing necessary business, or getting around for other purposes).

The smoking history by 5-year gender-specific birth cohort (e.g., 1885–1889, 1890–1894) is reconstructed based on the data in Burns et al. (1998). Their original cohort smoking history is estimated using 15 NHIS surveys between 1965 and 1991 [44]. The NHIS surveys collect information on respondents’ smoking status. Those who had smoked over 100 cigarettes in their life and were smoking every day or some days at the time of survey were defined as current smokers. This data is further updated by Preston et al. [45] using additional NHIS surveys through 2009, and converted into an estimate of the average number of years a cohort had smoked prior to age 40, which is also the smoking covariate used in the present study. This specific construction of smoking covariate is used because previous studies find a strong effect of cohort smoking history by middle age on health outcomes at older ages, and smoking duration is found to be a stronger predictor than intensity [25, 41, 46, 47].

Similarly, the variable for obesity is constructed in a cohort fashion as well. Obesity prevalence at age 40 is computed for each 5-year birth cohort by sex using NHIS data. Respondents’ height and weight are reported in NHIS surveys, and are then converted to Body Mass Index (BMI). Obesity is defined as having a BMI that is over 30 kg per square meter. Age 40 is also chosen for the construction of the obesity covariates, because middle age obesity has shown strong association with many chronic diseases in later life that cause disability and deaths [48–50].

In order to extrapolate the mean cumulative years of smoking by age 40 for cohorts that are still below 40 years old by 2010, a cohort’s mean cumulative years of smoking by age 40 is regressed on the observed mean cumulative years of smoking by age 35, by age 30, and by age 25 for the cohorts for which this information is all available. Similarly, a cohort’s prevalence of obesity at age 40 is regressed on the observed prevalence at age 35, at age 30, and at age 25 for those cohorts that have complete BMI information up to age 40. Dummy variables for sex and birth cohorts are added to these regressions. For smoking, the above models explain at least 97 % of the variance in the dependent variable in all cases. For obesity, over 92 % of the variance is explained. The corresponding values for the smoking and obesity variables is then estimated based on the coefficients estimated in the regression models. Because the end of the forecast period is 2040, the youngest cohort that requires extrapolation for the smoking and obesity variables are born in the years 1985–1989 and will reach 55 years old by 2040. However, the obesity variable also needs to be extrapolated back for cohorts born before 1935, as body weight information is collected only after 1976. This variable is only extrapolated back to cohorts born in 1920–1924, and is fixed at this level for cohorts born prior to 1920.

## Methods

### Estimating the transition rates

Three health states (non-disabled, disabled, dead) are considered in this study. Accordingly, there are four possible types of transitions: a healthy person may experience onset of disability, or may die; and a disabled person may recover, or die. The age-specific transition rates among the three health states are estimated using the multi-state life table (MSLT) approach proposed by Majer et al. [40]. The estimation is essentially based on the fact that the prevalence of disability for a cohort aged *x* + 1 at time *t* + 1 is a function of the following: prevalence of disability for the same cohort when it was aged *x* at time *t*, the probability of disability onset and recovery, as well as the probability of death for both non-disabled and disabled during this one-year time interval [40, 51]. However, for simplicity of modeling and to obtain more robust forecast, the recovery from disability is assumed to be absent and the relative risk of disability on mortality is constant over time and age, as in Majer et al. [40]. The details of this estimation method are discussed in the Additional file 1.

### Modeling and forecasting the transition rates

I assume the variations in the estimated transition rates for both mortality and disability can be partially explained by age and period [52]. The portion, other than the residual, that is left unexplained by age and period is considered to be influenced by the history of smoking and obesity [41]. Accordingly, the Lee-Carter model used incorporates cohort smoking and obesity history to fit and forecast all three types of transition rates. Since the two leading risk factors of mortality and morbidity are adjusted for, the temporal trends in mortality and morbidity are assumed to be the same for both sexes [41]. The model can be expressed as:

1 $$ \ln {m}_{x,t}^{\kern0.04em g,i}={\alpha}_x^{g,i}+{\beta}_x^{g,i}{\kappa}_t^i+{\theta}^{\kern0.04em g,i}{S}_{t-x}^g+{\lambda}^{g,i}{O}_{t-x}^g+{\varepsilon}_{x,t}^{\kern0.04em g,i} $$

where *g* specifies gender and *i* specifies the three types of transition: non-disabled to disabled (HU), non-disabled to death (HD), and disabled to death (UD). The parameter *α*_*x*_ is the average of the log transition rate at age *x* over time, *κ*_*t*_ quantifies the underlying development of transition rates over time, and is assumed to be the same for men and women when smoking and obesity are adjusted for. *β*_*x*_ is the changes in transition rates at age *x* in response to changes in *κ*_*t*_ over time. *S*_*t* − *x*_ and *O*_*t* − *x*_ are respectively cohort history of smoking and obesity for a cohort born in year *t-x*, and θ and λ are corresponding coefficients that measure the effect of smoking and obesity on the specific transition rates.

The parameters are estimated by minimizing the sum of squared errors of the singular value decomposition performed for both sexes combined, as specified by the following equation [42]:

2 $$ \left[\begin{array}{c}\hfill \ln {m}_{x,t}^{M,i}-{\theta}^{\kern0.04em M,i}{S}_{t-x}^M-{\lambda}^{M,i}{O}_{t-x}^M\hfill \\ {}\hfill \ln {m}_{x,t}^{F,i}-{\theta}^{F,i}{S}_{t-x}^F-{\lambda}^{F,i}{O}_{t-x}^F\hfill \end{array}\right]=\left[\begin{array}{c}\hfill {\alpha}_x^{M,i}\hfill \\ {}\hfill {\alpha}_x^{F,i}\hfill \end{array}\right]+{\kappa}_t^i\left[\begin{array}{c}\hfill {\beta}_x^{M,i}\hfill \\ {}\hfill {\beta}_x^{F,i}\hfill \end{array}\right]+\left[\begin{array}{c}\hfill {\varepsilon}_{x,t}^{M,i}\hfill \\ {}\hfill {\varepsilon}_{x,t}^{F,i}\hfill \end{array}\right] $$

In order to find a model that best fits the actual transition rates, the model specified in Eq. (1) is tested using different sets of covariates. Specifically, the model is run with no covariates as the conventional Lee-Carter model; with only cohort smoking history; with only cohort obesity history; with both cohort smoking and obesity history; and with both cohort smoking and obesity history as well as their interaction. The model that includes both cohort smoking and obesity history and their interaction is selected for forecasting both mortality and disability, based on its superior model fit statistics.

The random walk model with drift, or ARIMA (0, 1, 0) is used to produce future values of *κ*_*t*_ for years 2011 to 2040, as it yields reasonably good fit for all types of transitions. The variance-covariance matrix for *κ*_*t*_ of all three types of transitions is estimated to account for the future trends of these transitions jointly, and is used to produce 95 % confidence intervals for the projected transition rates and life expectancy through simulation. In the simulation, the distribution of the disturbances is assumed to be an independently and identically distributed multivariate normal distribution, which has a mean of zero and a covariance matrix identical to the variance-covariance matrix discussed above.

Then the future values of *k*, as well as corresponding cohort smoking and obesity history, are used to estimate the future transition rates from 2011 to 2040, which are eventually translated into disability-free life expectancy (LE^ND^) and life expectancy with disability (LE^D^) [53].

## Results

**Fig. 1:**
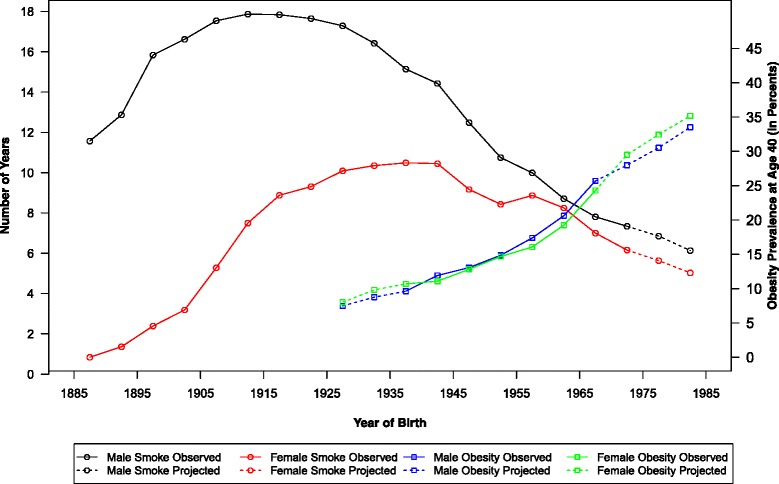
Smoking and obesity trends by birth cohorts

Figure 1 plots the trends of smoking and obesity by cohort. We see a rise in the average cumulative years a cohort had smoked by age 40 for both men and women among the earlier born cohorts and a decline among the younger cohorts. The peak is reached for the male cohorts born in 1910–1920 and the female cohorts born in 1935–1945 respectively. In contrast, both sexes have experienced continuous increases in the prevalence of obesity at age 40 for cohorts born after 1925. The values of both smoking and obesity variables are extrapolated for the youngest cohorts, for whom data are not yet available. In general, the declining smoking trend for cohorts born after 1970 and the increasing obesity trend for cohorts born after 1965 are preserved for the youngest cohorts.

**Fig. 2:**
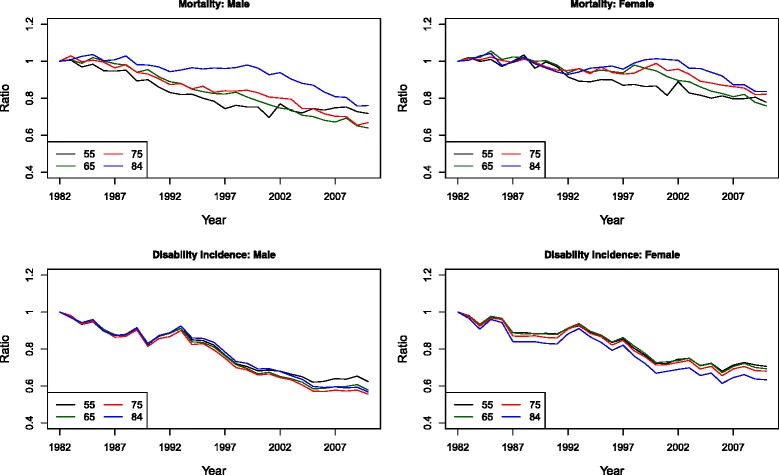
Ratios of observed transition rates over time (1982–2010) to observed rates in 1982

Figure 2 shows the trends of mortality and disability transitions over time by plotting the ratio of transition rates throughout the observation period (1982–2010) to those observed in 1982 at several ages (55, 65, 75, and 84). Because the relative risk of disability on mortality is assumed to be constant at all ages, the ratios for mortality of disabled and those for mortality of non-disabled are identical. Therefore, only the ratios for overall mortality are plotted. It is evident that men at all ages have experienced larger reductions in both mortality and disability than women during the entire observation period, reflecting men’s earlier decline in smoking [41, 45, 54].

Table 1 presents the results from fitting the modified Lee-Carter models to the three types of transition rates with different sets of covariates for both sexes. Model 1 is simply a Lee-Carter model without any covariates. Model 2 includes cohort smoking history only, while Model 3 includes cohort obesity history only. In Model 4, both smoking and obesity covariates are included. Model 5 additionally includes an interaction term of smoking and obesity. Due to the constant assumption for the relative mortality risk of being disabled, the estimates for mortality of disabled and of non-disabled are the same for all models. In general, Model 5 performs best based on its larger adjusted R-square and hence selected for the forecasting model in this analysis. Additional file 2 provides detailed discussions for the model selection.

**Table 1:** Parameter estimates for smoking and obesity from different specifications of the Lee-Carter model

		Mortality					Net disability incidence				
		Model 1	Model 2	Model 3	Model 4	Model 5	Model 1	Model 2	Model 3	Model 4	Model 5
Male	Smoking	-	0.0266	-	0.0263	0.0668	-	0.0078	-	0.0036	0.0255
	Obesity	-	-	−0.0129	−0.0077	0.0674	-	-	−0.005	−0.0035	0.0289
	Interaction	-	-	-	-	−0.0042	-	-	-	-	−0.0018
Female	Smoking	-	0.0222	-	0.0161	0.033	-	−0.0002	-	0.0006	0.0088
	Obesity	-	-	−0.0038	−0.0012	0.0115	-	-	0.0069	0.0068	0.0181
	Interaction	-	-	-	-	−0.001	-	-	-	-	−0.0011
R-Square		0.9461	0.9528	0.9311	0.9494	0.9597	0.9884	0.9881	0.9889	0.9888	0.9895

**Fig. 3:**
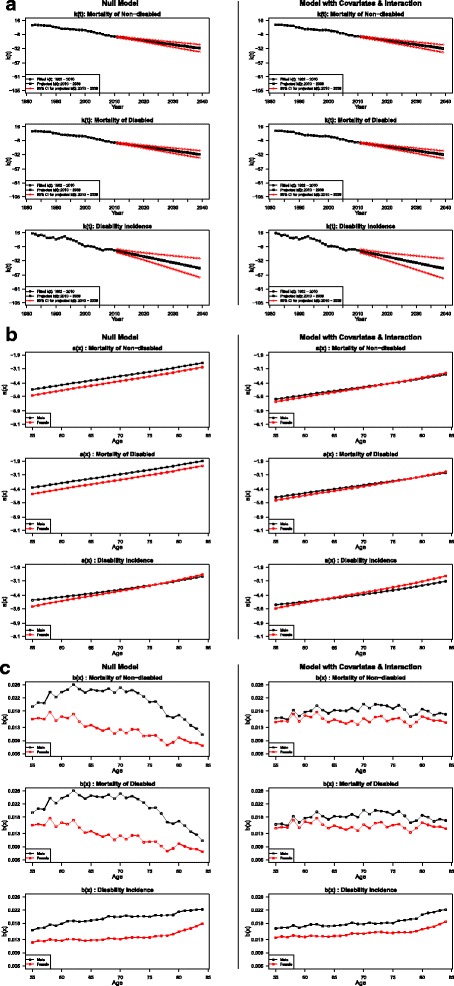
**a** Estimates and projections for k(t) from both the conventional and modified Lee-Carter model. **b** Estimates for a(x) from both the conventional and modified Lee-Carter model. **c** Estimates for b(x) from both the conventional and modified Lee-Carter model

Figure 3a–c, respectively, present the estimates for k(t), a(x) and b(x) in both the conventional and modified Lee-Carter models. Estimates from the conventional Lee-Carter model (Model 1) are shown in panels on the left, and estimates from the selected model for forecasting (Model 5) are shown in panels on the right. Again, due to the assumption of a constant impact of disability on mortality, the estimates of k(t) and b(x) are identical for mortality for disabled and non-disabled, although the estimates for a(x) for these two types of transition differ. The addition of covariates and interaction does not lead to substantial change in k(t), as shown in Fig. 3a. Nevertheless, when k(t) is multiplied by b(x) and then added to a(x) which both change substantially due to different model specifications, variations in transition rates and their translations into healthy life expectancy can still be striking. In Fig. 3b, the comparison of the plots of a(x) for mortality demonstrates that the inclusion of the two covariates and their interaction explains a great proportion of the gender-difference in the underlying mortality profile by age. Furthermore, in the left panel of Fig. 3b, the underlying disability incidence rates at younger ages are higher for men than for women, reflecting the greater reduction in cumulative smoking history for men at younger ages, which results in a higher survival of the already disabled or potentially disabled population. This pattern largely disappears once covariates and interaction are included, and the underlying disability incidence appears to be higher for women particularly at older ages, consistent with previous epidemiologic studies that find women are more vulnerable to disabling conditions such as fractures, osteoarthritis, and back problems [16, 17, 55].

Similar to the findings in Wang and Preston [41], when smoking and obesity are not adjusted for in the Lee-Carter models, the disparities in the b(x) estimates between men and women are evident for both mortality and disability (left panel of Fig. 3c). Once smoking and obesity are included, the disparities become much smaller and the slopes of the age pattern of change in transition rates become more level, as shown in the right panel in the figure. Specifically, the b(x) estimates for mortality for men and women show less distorted pattern. Similarly, those estimates for disability for men and women are more parallel and their differences are reduced by approximately 40 %. Moreover, for younger ages, the age-specific mortality change in response to the temporal trend of mortality change is larger than for older ages in the left panel, but smaller in the right panel. Since the younger cohorts have experienced more remarkable declines in smoking but increases in obesity, the above result indicates that adjusting for the time trends of smoking and obesity produces a less distorted age pattern of mortality change. Also, the impacts of smoking decline are more salient than the impacts of obesity increase.

**Fig. 4:**
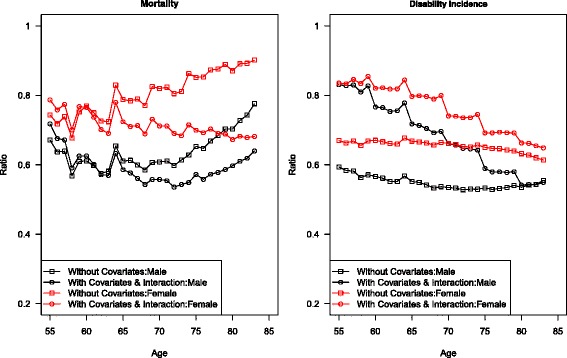
Ratios of projected transition rates in 2040 to transition rates observed in 2010

To demonstrate the impact of smoking and obesity on the projections of future mortality and morbidity, the ratio of projected transition rates at 2040 is compared to those observed at 2010 for the null model and the final model in Fig. 4. Because the impact of disability on mortality is assumed to be constant over age, only the graph for mortality of non-disabled is shown. The ratios of mortality estimated using both model specifications for women are greater than the ratios for men, reflecting men’s sharper decline in smoking among these cohorts. Inclusion of covariates leads to lower mortality as expected. For both sexes, the differences in ratios for mortality estimated with and without covariates are greater at older ages, as the cohorts that will reach older ages by the end of the projection period are the ones who have experienced the largest smoking decline. Contrarily, inclusion of covariates leads to higher disability incidence. And the differences in ratios for disability estimated with and without covariates are greater at younger ages, reflecting the fact that the obesity epidemic is more recent. This may also suggest that in the future, disability is more likely to be attributable to obesity than to smoking.

Furthermore, model selection has a greater impact on mortality projection for women but a greater impact on disability projection for men. Given that men and women have similar patterns in cohort obesity history, the difference in projections due to different model selection seems to originate from the gender difference in smoking history. The larger impact of model selection on mortality projection for women, particularly at older ages, is consistent with the timing of extinction of the heaviest smoking female cohorts. Similarly, the disability projection for men is more sensitive to model selection, particular at younger ages, because men’s extended trend of smoking decline has provided relatively larger exposure for the disabling effect of obesity to operate.

**Fig. 5:**
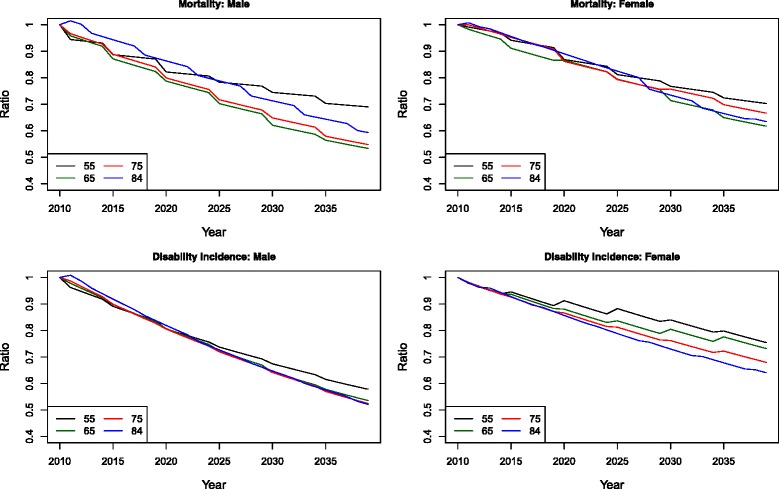
Ratios of transition rates over time for the forecasting period (2010–2040) to observed rates in 2010

Figure 5 presents the projected mortality and disability transition rates over time relative to the observed ones in 2010, given the cohort history of smoking and obesity. Overall, all age groups for both sexes will experience decline in mortality and morbidity. The oldest cohort in this figure was born in 1925–1929, which is younger than the heaviest smoking male cohort but older than the heaviest smoking female cohort. Therefore, the mortality associated with smoking will decline steadily for men across all cohorts. However, the younger cohorts, particularly for those born after 1955 (aged 55 in 2010), have substantially higher prevalence of obesity that offsets the smoking-related mortality declines. Thus, a crossover is seen on the graph for male mortality. The decline in smoking for women, on the other hand, only occurred for cohorts born after 1945. Consequently, the pattern of mortality decline for women across cohorts is more complex, given different directions of change in smoking-related mortality and the increase of obesity prevalence as well as their interaction. Nevertheless, overall the obesity epidemic reduces the rates of mortality decline for women as well.

Besides mortality, male morbidity is also expected to decline for all age groups over time. Compared to the projected trends of male mortality and female morbidity, male morbidity shows less of a cohort pattern, except for cohorts born recently that have highest prevalence of obesity. This indicates that smoking is more likely to have a fatal rather than disabling effect for men, while obesity is disabling for men but the effect is not as strong for women. The decline in male morbidity is more likely to be attributable to improvement in medical care that affects the underlying morbidity profile at all ages. For women, a clear cohort pattern can be seen for all age groups. Additionally, this pattern is only observed among cohorts born after 1950, suggesting that this cohort pattern origins from the increase in obesity prevalence rather than from the change in smoking. It is, therefore, consistent with previous studies that argue obesity has stronger impact on female morbidity [16, 17, 55].

**Fig. 6:**
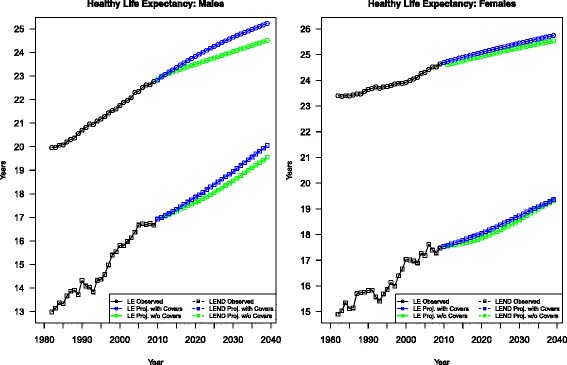
Observed and forecasted healthy life expectancy (LE and LE^ND^) using different models

Finally, the life expectancy (LE), disability-free life expectancy (LE^ND^), and life expectancy with disability (LE^D^) between age 55 and 85 are projected up to 2040 for both sexes, as shown in Table 2 and Fig. 6. In accordance with the findings in Crimmins et al. [56, 57], the US elderly have experienced substantial increases in both LE and LE^ND^ during the observation period from 1980s to 2010, and the increases in LE are mostly attributable to the increase in LE^ND^ along with the decrease in LE^D^, suggesting compression of disability [57, 58]. While this is true for both sexes, men appear to benefit from more years of gain in LE^ND^ than women.

**Table 2:** Life expectancy between age 55 and 85 by health status

		Males				Females			
	Year	LE	LE^ND^	LE^D^	LE^ND^/LE (%)	LE	LE^ND^	LE^D^	LE^ND^/LE (%)
Observed	1982	19.96	12.98	6.98	65.03	23.40	14.90	8.50	63.68
	1990	20.70	14.32	6.38	69.18	23.64	15.82	7.82	66.92
	2000	21.75	15.80	5.95	72.64	23.92	17.04	6.88	71.24
	2010	22.82	16.92	5.90	74.15	24.69	17.53	7.16	71.00
Projected without covariates	2020	23.53 (23.26, 23.77)	17.64 (17.45, 17.83)	5.89 (5.73, 6.02)	75.00 (74.57, 75.49)	24.92 (24.81, 25.03)	17.91 (17.81, 18.00)	7.01 (6.91, 7.10)	71.89 (71.56, 72.18)
	2030	24.06 (23.60, 24.43)	18.58 (18.21, 18.95)	5.48 (5.10, 5.80)	77.22 (76.14, 78.59)	25.25 (25.08, 25.43)	18.62 (18.37, 18.83)	6.64 (6.42, 6.90)	73.72 (72.79, 74.49)
	2040	24.54 (23.94, 25.01)	19.6 (19.04, 20.13)	4.94 (4.40, 5.45)	79.87 (78.11, 81.86)	25.55 (25.33, 25.77)	19.27 (18.96, 19.68)	6.18 (5.86, 6.59)	75.82 (74.36, 76.98)
Projected with covariates and interaction	2020	23.83 (23.58, 24.05)	17.86 (17.70, 18.03)	5.97 (5.81, 6.14)	74.95 (74.41, 75.45)	25.11 (24.95, 25.24)	18.00 (17.89, 18.13)	7.09 (6.99, 7.21)	71.72 (71.32, 72.08)
	2030	24.63 (24.24, 24.98)	18.99 (18.68, 19.36)	5.62 (5.30, 6.06)	77.17 (75.61, 78.38)	25.49 (25.23, 25.69)	18.67 (18.41, 18.97)	6.80 (6.54, 7.13)	73.28 (72.13, 74.23)
	2040	25.24 (24.75, 25.67)	20.10 (19.60, 20.64)	5.10 (4.65, 5.78)	79.79 (77.35, 81.53)	25.74 (25.40, 26.00)	19.30 (18.88, 19.77)	6.41 (6.00, 6.96)	75.03 (73.07, 76.53)

Conventional Lee-Carter modeling predicts continued gains in LE and LE^ND^ for men and women in the coming decades, although with decelerated rates of increase compared to the previous 30 years. Relative to 2010, men will have a 1.72 years gain in LE between age 55 and 85, whereas the figure for women is half of that. The gains in LE can be decomposed to about 1 year loss in LE^D^ for both sexes, and about 2.7 years and 1.7 years gain in LE^ND^ for men and women, respectively.

For men, the addition of cohort smoking and obesity history and their interaction yields even more optimistic projections than the model with no covariates. Relative to the null model, the final model projects an extra 0.30, 0.57, and 0.70 years gain in LE at 2020, 2030, and 2040 respectively, and an extra 0.22, 0.41, and 0.50 years gain in LE^ND^ at 2020, 2030, and 2040 respectively. This indicates that net of the increase in obesity prevalence, the decline in smoking still leads to progressive gain in life expectancy for American men over the next three decades, of which over 70 % is attributable to increase in disability-free life expectancy.

In contrast, including both covariates and their interaction produces a smaller increase of LE and LE^ND^ for women, mainly because of the slower improvement in survival produced by their lagged decline in smoking during the observation period. Compared to the null model, the final model only leaves women an additional 0.19, 0.24, and 0.19 years of LE at 2020, 2030, and 2040 respectively, and an additional 0.09, 0.05, and 0.03 years of LE^ND^ at 2020, 2030, and 2040 respectively. As the heaviest smoking female cohort reaches its prime age of death in 2020s, the decline in gains in LE and LE^ND^ adjustments over time relative to the null model can be best explained by the fact that obesity has large, destructive impact on women’s health.

Moreover, both sexes are expected to spend a larger proportion of their remaining life time disability-free. Relative to the model without covariates, however, the model proposed in this study produces only slightly smaller of this proportion for men throughout the projection period but an almost 1 % decrease for women by 2040. This indicates the impact of fall in smoking and the impact of rise in obesity prevalence tend to balance each other out for men in terms of quality of health, but for women the negative effect of rise in obesity will likely to outweigh the positive effect of fall in smoking in the next decades, confirming the findings in existing literature about the gender difference in the impacts on mortality and morbidity of both smoking and obesity.

### Model validation

In addition to measuring the goodness-of-fit for the forecasting model using R-squares, out-of-sample model validation is performed by holding out the data from 2001 to 2010 and comparing these data with 10-year projections made with data only from 1982–2000. For men, the maximum error relative to the observed value is roughly 0.22 years for LE, 0.35 years for LE^ND^, and 0.29 years for LE^D^. For women, it is 0.16 years for LE, 0.19 years for LE^ND^, and 0.23 years for LE^D^. In conclusion, these results indicate the forecasting model is valid and generalizable for data from varying periods.

### Sensitivity analyses

The NHIS data used in this study is limited to the non-institutionalized population which is presumably healthier than the institutionalized population. Therefore, the net disability transition rate tends to be underestimated. The magnitude of this underestimation is evaluated by performing the analysis with additional data from the American Community Survey (ACS) from 2006 to 2010 for the institutionalized population (people who live in correctional institutions, mental institutions, or institutions for the elderly or handicapped). The ACS is an ongoing, mandatory statistical survey covering a small percentage of the US population every year. 1 % of the population, including institutionalized persons, were randomly sampled in 2006–2010. As in the main analysis, only older adults aged between 55 and 85 years are included by 5-year age group. Disability is defined as individuals having a condition that substantially limits their basic physical activities (e.g., walking, climbing stairs, reaching, lifting, or carrying); or having any physical, mental, or emotional condition lasting more than half a year that limit their ability taking care of their own personal needs (e.g., bathing, dressing, or getting around inside the home) or activities outside the home alone. Accordingly, these limitations are comparable to ADLs and IADLs in NHIS in the main analysis. In addition to variables indicating whether a respondent has limitations in activity, a variable that indicates whether one resides in institutions is available from 2006 to 2010. The prevalence of disability was calculated for the entire population using this information and the forecasting model was re-run. Relative to the life expectancy measures for 2006–2010 calculated in this practice, using data for only non-institutionalized population overestimates the LE by a maximum of 0.002 years and the LE^ND^ by a maximum of 0.15 years, but underestimates the LE^D^ by a maximum of 0.15 years for men. For women, the LE and the LE^ND^ are overestimated by a maximum of 0.003 years and 0.26 years, and the LE^D^ is underestimated by a maximum of 0.26 years. Moreover, the estimates used from this analysis project the LE^ND^ and LE^D^ for 2011–2015 and these projections are compared with those from projections based on NHIS surveys in 2006–2010. If only data for the non-institutionalized population are used, the projections for the LE and the LE^ND^ in 2011–2015 are overestimated by a maximum of 0.03 years and 0.14 years respectively, but the projection for the LE^D^ is underestimated by a maximum of 0.12 years. For women, the LE and the LE^ND^ are overestimated by a maximum of 0.003 years and 0.18 years respectively, but the projection for the LE^D^ is underestimated by a maximum of 0.18 years. Therefore, the effect of excluding the institutionalized population from the estimates and projections can be considered small.

Furthermore, the proposed model is subject to two assumptions: 1) the impact of disability on mortality is constant over age, and 2) there is no recovery from being disabled. Two additional sensitivity analyses are performed to test the robustness of the model, by estimating the effects of violation of these two assumptions on age-specific transition rates and its aggregated effects on healthy life expectancy between age 55 and 85.

As suggested in Guillot and Yu [51], both the relative mortality risk of disability and the probability of recovery are modeled as exponential functions of age as below:

$$ H{R}_x={\alpha}_1{e}^{\beta_1x} $$

$$ {q}_x^{UH}={\alpha}_2{e}^{\beta_2x} $$

The parameter estimates from Guillot and Yu [51] is used for the values of *α*_1_ (5.51), *β*_1_ (−0.049), *α*_2_ (0.353), and *β*_2_.(−0.043). These parameters are estimated for men and women combined based on data from Health and Retirement Study (HRS) 1998 and 2000 with a transformed age *a = x-*65. Results of projected healthy life expectancy at 2010, 2020, 2030, and 2040 are shown in Table 3 and Table 4.

**Table 3:** Sensitivity of results to the impact of disability on mortality

	Males			Females		
	LE	LE^ND^	LE^D^	LE	LE^ND^	LE^D^
2010	−0.02	0.01	−0.03	0.02	0.02	0
2020	−0.01	0.07	−0.06	−0.01	0.01	−0.03
2030	0	0.12	−0.12	−0.03	0.1	−0.13
2040	0.02	0.15	−0.13	−0.02	0.18	−0.2

A positive value means that the alternative assumption resulted in a gain in life expectancy relative to the main model

**Table 4:** Sensitivity of results to recovery

	Males			Females		
	LE	LE^ND^	LE^D^	LE	LE^ND^	LE^D^
2010	0.05	0.11	−0.06	0.04	0.12	−0.08
2020	0.03	0.12	−0.09	0.02	0.1	−0.08
2030	0.1	0.14	−0.04	0.08	0.1	−0.02
2040	0.07	0.2	−0.13	0.08	0.14	−0.06

A positive value means that the alternative assumption resulted in a gain in life expectancy relative to the main model

When it is modeled as an exponential function, the mortality risk of being disabled can be as high as 8.98 times and as low as 2.06 times of the mortality risk of being healthy at age 55 and 85 respectively. Once translated into healthy life expectancy, however, the effect of violation of the constant impact of disability on mortality assumption produces only small changes, as shown on Table 3. Overall, increase in the relative mortality risk of disability leads to only 0.15 years increase in LE^ND^, 0.13 years decline in LE^D^ and hence 0.02 years increase in LE for males by the end of the projection period. Similarly, the corresponding changes for females are 0.18 years increase in LE^ND^, 0.2 years decrease in LE^D^, and 0.02 years decreases in LE in 2040.

Neither does the inclusion of recovery from being disabled result in substantial changes in the projection of future healthy life expectancy. Table 4 shows that including recovery in the model yields gain in LE^ND^ and loss in LE^D^ for both sexes. The maximum gain in LE^ND^ is 0.14 years for both men and women, while the maximum loss in LE^D^ is 0.13 years and 0.08 years for men and women respectively. Overall, the gain in LE^ND^ and loss in LE^D^ offset each other, and hence lead to respectively 0.1 years and 0.08 years gain in LE for men and women.

## Discussion

Throughout the 20th century, the prevalence of cigarette smoking in the US can be best described as an inverse U-shaped curve, with the presence of sex difference [45, 54]. In contrast, the prevalence of obesity has shown an upward trend in recent decades [6, 7, 28]. To the best of my knowledge, this study is the first to use summary demographic measures (LE^ND^ and LE^D^), in association with observed and projected trends of smoking and obesity, to assess the health quality of the US older population in the past and future. The results reveal that men and women are both expected to have rising LE and LE^ND^ as well as falling LE^D^ between 2010 and 2040, resulting in compression of disability. Estimates from our model suggest that a large proportion of the difference in mortality and disability between men and women can be attributed to their different smoking patterns and the gender difference in the impacts of smoking and obesity. Specifically, men will benefit more from their earlier decline in smoking and have larger gain in LE than women, narrowing the gender gap in the current LE down to 0.5 years by 2040. The combined effects of existing and expected change in smoking and obesity will also lead to more years living without disability and fewer years living with disability. Men are projected to have a 3.2 years increase in LE^ND^ over the 30-year forecasting period, almost twice the gain for women. Besides men’s advantage in LE due to an earlier start in smoking decline, this difference in LE^ND^ may as well be partially attributed to the greater impact of obesity on disability for women, which offsets some of the gains in LE^ND^ produced by the smoking decline.

Notwithstanding the difference in models, definitions of healthy life expectancy, and the additional projection elements, results from this analysis are consistent with the trends in quality-adjusted life expectancy (QALE) observed in Stewart et al. [58]. In general, the climbing obesity rates decelerated the gain in QALE as well as the morbidity compression from 1987 to 2008, and the effect is fairly pronounced among older age groups. Also, whereas female survivors have higher morbidity, the gender gap is closing.

A major strength of this study is the inclusion of cohort smoking and obesity history, which largely explain the variations in mortality and morbidity. Without these variables, the projections will likely underestimate the future decline in mortality mainly due to ignoring the downward trend of smoking, but overestimate the decline in morbidity mainly due to ignoring the upward trend of obesity. In addition, because the cohort-based information for majority of the population that reach 55 in the projection period is already observable, this method only requires few extrapolation for the covariates and therefore produces more reliable projections than period-based methods.

Nevertheless, this analysis inevitably faces several limitations. First, the NHIS data used in this study is limited to the non-institutionalized population which is presumably healthier than the institutionalized population. Therefore, the net disability transition rate tends to be underestimated. Second, the proposed model is subject to two assumptions: 1) the impact of disability on mortality is constant over age, and 2) there is no recovery from being disabled. Both assumptions may not precisely reflect the reality. The above two limitations are addressed by performing sensitivity analyses, using alternative data and models [51]. The results suggest that in general neither limitation yields substantially different estimates and projections. Third, the continuing medical advances in the future may alter the relationship between smoking and obesity and health outcome, and hence the forecasts in this study may overestimate the future mortality and morbidity. However, as these medical improvements presumably apply to the whole population in general and their past trends are captured in the period parameter k(t) based on which the forecasts are produced, our forecasts take into account the corresponding uncertainty and reflect it in the prediction intervals.

Moreover, although our estimates of healthy life expectancy during the observed period are consistent with other studies [56–58] and the model validation demonstrates reasonably good forecasting performance, our forecasts are not free from model-based uncertainty due to the selection of only one of many potential models with different specifications, such as functional form, covariates, and lag time. Since an extensive search for alternative models is not the primary goal of this study and may introduce selection bias, addressing model-based uncertainty for forecasting healthy life expectancy using appropriate methodologies is worth pursuing in future research. In addition, the BMI variable and disability variable are constructed using self-reported data. However, prior studies have found overall strong concordance between self-reported and clinically documented health-related data [59–62]. Also, as this study accounts for only one dimension of smoking (cumulative duration) and obesity (prevalence), future research could attempt to use more comprehensive measurements that accounts for multiple aspects of smoking and obesity.

## Conclusion

In conclusion, this study confirms existing literature that smoking and obesity both have independent negative influences on individuals’ health, including both survival and activities. Operating jointly, they unanimously raise mortality for both disabled and non-disabled. However, because the incidence and prevalence of disability depend on survival which is affected by the two risk factors in a different direction, their interplay will likely yield different patterns of morbidity for men and women, due to their different smoking history and women’s vulnerability to the detrimental effects of obesity. Given the current state of epidemiologic transition, extra efforts should be directed to sustainable reduction in smoking, reversing the obesity epidemic and female morbidity.

## Abbreviations

HMD, Human Mortality Database; LE, life expectancy; LE^D^, life expectancy with disability; LE^ND^, disability-free life expectancy; MSLT, multi-state life table; NHIS, National Health Interview Survey

## Additional files

Additional file 1: Estimating the transition rates. (DOCX 74 kb) Additional file 2: Selecting the forecasting model. (DOCX 22 kb)
