# Supplementary material for: Future healthy life expectancy among older adults in the US: a forecast based on cohort smoking and obesity history
Source: Popul Health Metr. 2016 Jul 12;14:23. doi: 10.1186/s12963-016-0092-2 (PMC4941025; doi:10.1186/s12963-016-0092-2)
Supplement: Additional file 1: — Estimating the transition rates. (DOCX 74 kb) [file 12963_2016_92_MOESM1_ESM.docx]

**Supplement File I: Estimating the transition rates**

Three health states (non-disabled, disabled, dead) are considered in this study. Accordingly, there are four possible types of transitions: a healthy person may experience onset of disability, or may die; and a disabled person may recover, or may die. As Guillot and Yu (2009) and Majer et al. (2013) both point out, the prevalence of disability for a cohort aged *x*+1 at time *t*+1 is a function of the following: prevalence of disability for the same cohort when it was aged *x* at time *t*, the probability of disability onset and recovery, as well as the probability of death for both non-disabled and disabled during this one-year time interval^1,2^. This can be expressed by the equation below.

$\pi_{x+1,t+1}^{U}=\frac{\pi_{x,t}^{U}(1-q_{x,t}^{UD}-q_{x,t}^{UH})+(1-\pi_{x,t}^{U})q_{x.t}^{HU}}{1-(1-\pi_{x,t}^{U})q_{x,t}^{HD}-\pi_{x,t}^{U}q_{x,t}^{UD}}$ (1)

where $\pi_{x+1,t+1}^{U}$ and $\pi_{x,t}^{U}$ are the corresponding prevalence of disability for a cohort aged *x*+1 at time *t*+1 and for the same cohort exactly a year ago. These prevalence rates are estimated using NHIS surveys conducted in two consecutive years, using NHIS sample weights to account for non-responses and make prevalence rates representative for the US non-institutionalized population.

$q_{x,t}^{HD}$ and $q_{x,t}^{UD}$ respectively denote the probability that non-disabled and disabled individuals aged *x* at time *t* will die between time *t* and *t*+1. These probabilities can be separately derived from the mortality rates of non-disabled and disabled. Let $M_{x,t}$ be the matrix of the instantaneous transition rates between different health states at age *x* and time *t*. The element in the *i*th row and *j*th column therefore indicates the transition rate that an individual aged *x* at time *t* in the *i*th state will move into the *j*th state instantaneously. For this particular study,

Similarly, the matrix of the probability an individual aged *x* at time *t* in the *i*th state will transit into the *j*th state within a one-year interval can be written as:

Assuming the distributions of the transitions are linear with age and all transitions occur at exact the middle of the one-year interval, a relationship between $M_{x,t}$ and $Q_{x,t}$ can be established,

$$Q_{x,t}=\frac{I-\frac{M_{x,t}}{2}}{I+\frac{M_{x,t}}{2}}$$

where *I* is an identity matrix.

The transition probabilities between specific health states can be converted as below, given the assumption of no recovery from disabled to non-disabled,

$$q_{x,t}^{HU}=\frac{m_{x,t}^{HU}}{(1+\frac{m_{x,t}^{HU}}{2}+\frac{m_{x,t}^{HD}}{2})(1+\frac{m_{x,t}^{UD}}{2})}$$

$$q_{x,t}^{HD}=\frac{m_{x,t}^{HU}+m_{x,t}^{HD}}{(1+\frac{m_{x,t}^{HU}}{2}+\frac{m_{x,t}^{HD}}{2})}-q_{x,t}^{HU}$$

However, no separate age-specific mortality rates are available for disabled and non-disabled, because of the lack of a longitudinal dataset that is large enough to provide reliable estimates for multiple age groups in a long period, as needed for the forecasting purpose in this study. Therefore I take advantage of the following inter-relationship among overall mortality ($m_{x,t}$), state-specific mortality ($m_{x,t}^{HD}$and $m_{x,t}^{UD}$), relative mortality risk of disabled ($HR_{x,t}$), and prevalence of disability ($\pi_{x,t}^{U}$) to derive separate mortality rates for disabled and non-disabled:

$m_{x,t}^{UD}=m_{x,t}^{HD}HR_{x,t}$ (2)

and $m_{x,t}^{HD}=\frac{m_{x,t}}{HR_{x,t}\times\pi_{x,t}^{U}+(1-\pi_{x,t}^{U})}$ (3)

The only unknown in equation (3) is the hazard ratio of disabled. This is estimated using individual-level data from NHIS conducted in 1986-2004, during which period each participant in the survey is linked to death certificate data found in the National Death Index (NDI). A Cox proportional hazard model with left truncation is fitted by sex, and no significant age or year interaction is found^3^. Consequently, I assume the estimated hazard ratios, 2.10 (95% confidence interval [CI]: 2.07-2.13) for men and 2.02 (95% CI: 1.99-2.05) for women, are constant over time and age.

Now only the probability of disability onset ($q_{x.t}^{HU}$) and the probability of recovery from disability ($q_{x.t}^{HU}$) in equation (1) are left unsolved. For simplicity of modeling and to obtain more robust forecast, I assume the recovery from disability is absent, as practiced in Majer et al. (2013). As a result, the transition from non-disabled to disabled can be considered as the net incidence of disability, and equation (1) can be re-written as below and can be used to derive the only unknown$q_{x.t}^{HU}$:

$\pi_{x+1,t+1}^{U}=\frac{\pi_{x,t}^{U}(1-q_{x,t}^{UD})+(1-\pi_{x,t}^{U})q_{x.t}^{HU}}{1-(1-\pi_{x,t}^{U})q_{x,t}^{HD}-\pi_{x,t}^{U}q_{x,t}^{UD}}$ (4)

Once the probability of transitions between different states are estimated, the life expectancy with or without disability can be calculated using multi-state life table method^4^. Let $l_{x,t}$denote the matrix of number of persons alive at age *x* and time *t* in different health states, and $l_{x,t}=\left[\begin{matrix} l_{x,t}^{H} \\ l_{x,t}^{U} \end{matrix}\right]$.

Accordingly, the number of people alive one year later is

Next, person-years lived by people aged *x* alive at time *t* by health status can be estimated by

$$L_{x,t}=\left[\begin{matrix} \frac{l_{x,t}^{H}+l_{x+1,t+1}^{H}}{2} \\ \frac{l_{x,t}^{U}+l_{x+1,t+1}^{U}}{2} \end{matrix}\right]$$

The cumulative persons-years lived by individuals aged *x* alive at time *t* is

$$T_{x,t}=\left[\begin{matrix} T_{x,t}^{ND} \\ T_{x,t}^{D} \end{matrix}\right]=\sum_{a=x}^{85} L_{a,t}$$

Finally, LE^ND^ and LE^D^ as well as the total LE at age *x* and time *t* are calculated as below

$$LE_{x,t}^{ND}=\frac{T_{x,t}^{ND}}{l_{x,t}}$$

$$LE_{x,t}^{D}=\frac{T_{x,t}^{D}}{l_{x,t}}$$

$$LE_{x,t}=LE_{x,t}^{ND}+LE_{x,t}^{D}$$

**References**

1. Guillot M, Yu Y. Estimating health expectancies from two cross-sectional surveys: The intercensal method. *Demogr Res*. 2009;21:503-534.

2. Majer IM, Stevens R, Nusselder WJ, Mackenbach JP, van Baal PHM. Modeling and forecasting health expectancy: theoretical framework and application. *Demography*. 2013;50(2):673-97. doi:10.1007/s13524-012-0156-2.

3. Cox DR. Regression models and life tables. *J R Stat Soc Ser B*. 1972;34(2):187-220.

4. Preston SH, Heuveline P, Guillot M. *Demography: Measuring and Modeling Population Processes*. (Heuveline P, Guillot M, eds.). Blackwell; 2001. doi:10.2307/1535065.
