# Supplementary material for: Future healthy life expectancy among older adults in the US: a forecast based on cohort smoking and obesity history
Source: Popul Health Metr. 2016 Jul 12;14:23. doi: 10.1186/s12963-016-0092-2 (PMC4941025; doi:10.1186/s12963-016-0092-2)
Supplement: Additional file 2: — Selecting the forecasting model. (DOCX 22 kb) [file 12963_2016_92_MOESM2_ESM.docx]

**Supplement File II: Selecting the forecasting model**

Table 1 presents the results from fitting the modified Lee-Carter models to the three types of transition rates with different sets of covariates for both sexes. Model 1 is simply a Lee-Carter model without any covariates. Model 2 includes cohort smoking history only, while Model 3 includes cohort obesity history only. In Model 4, both smoking and obesity covariates are included. Model 5 additionally includes an interaction term of smoking and obesity. Due to the constant assumption for the relative mortality risk of being disabled, the estimates for mortality of disabled and of non-disabled are the same for all models.

When I only adjust for smoking in the Lee-Carter model, a negative effect of smoking on survival is observed for both men and women, with the smoking risk for men being higher than that for women. This is consistent with existing literature that finds men are more responsive to the adverse effect of smoking than women^1,2^. However, obesity is found to be associated with lower mortality for both sexes (especially for men) when it is the only covariate included. As Figure 2 shows, the rise in cohort obesity prevalence is accompanied by the fall of cumulative smoking duration, to which the mortality decline in recent years is mainly attributed, particularly for men. Consequently, omitting smoking in the model leads to these counterintuitive estimates and smaller adjusted R-square in Model 3.

Adjusting for both covariates simultaneously to some extent reduces the confounding introduced by smoking. Since at the individual level smokers are on average leaner and less healthy, smoking decline tends to lead to higher obesity prevalence at the aggregated level. This may explain why the impact of obesity is still confounded and appears to be positive in Model 4. In Model 5, an interaction of smoking and obesity is added. Both smoking and obesity are now found to be associated with higher mortality and interact with each other negatively. This is consistent with findings in the literature^3,4^. Although the effects of smoking and obesity are not directly comparable using the coefficient estimates because of their different metrics, it is possible to compare the individual impact of smoking and obesity on men relative to women. For mortality, the coefficient of the smoking covariate for men is almost twice of that for women, the coefficient of obesity for men is nearly six times of that for women, and the coefficient of interaction for men is four times of that for women. This suggests men are more responsive to the impact of smoking and obesity on mortality even when interaction is taken into account, confirming the gender difference arguments in existing studies^1,5^.

In contrast, despite the small magnitude, smoking alone is shown to increase the net disability transition rate for men but decrease it for women, while the signs are reversed for the effects of obesity in Model 3 just as in the models for mortality. This is likely because for men, the past decline in smoking has averted more disability incidence than the rise in obesity prevalence has caused, but for women, it is the opposite as a result of both the delayed trend of smoking and obesity’s greater disabling impact for women^6–8^. Including both covariates only partially removes the bias, while adding the interaction term produces results suggesting that both smoking and obesity are associated with higher risks of becoming disabled. Furthermore, compared to the estimates from Model 5 for female mortality, the estimates from Model 5 for female disability yield a larger effect for obesity but a disproportionately smaller effect for smoking, while the effects for interaction are similar, suggesting that obesity contributes to a greater proportion of female disability than to female mortality. For men, in contrast, the relative impacts of smoking and obesity are roughly the same for both mortality and disability.

Overall, the addition of both smoking and obesity covariates and their interaction explains respectively 25% and 10% of the variances of mortality and disability that are otherwise left unexplained by simple Lee-Carter model with no covariates. Specifically, the adjusted R-squares increase from 0.9461 to 0.9597 for mortality and from 0.9884 to 0.9895 for disability. Accordingly, projections of the transition rates are made based on the relationship discovered in Model 5 for both mortality and disability.

**References**

1. Chao A, Thun MJ, Henley SJ, Jacobs EJ, McCullough ML, Calle EE. Cigarette smoking, use of other tobacco products and stomach cancer mortality in US adults: The Cancer Prevention Study II. *Int J Cancer*. 2002;101(4):380-389. doi:10.1002/ijc.10614.

2. Wang H, Preston SH. Forecasting United States mortality using cohort smoking histories. *Proc Natl Acad Sci U S A*. 2009;106(2):393-8. doi:10.1073/pnas.0811809106.

3. Koster A, Leitzmann MF, Schatzkin A, et al. The combined relations of adiposity and smoking on mortality. *Am J Clin Nutr*. 2008;88(5):1206-1212.

4. Krueger PM, Rogers RG, Hummer RA, Boardman JD. Body Mass, Smoking, and Overall and Cause-Specific Mortality Among Older U.S. Adults. *Res Aging*. 2004;26(1):82-107.

5. Finkelstein EA, Brown DS, Wrage LA, Allaire BT, Hoerger TJ. Individual and aggregate years-of-life-lost associated with overweight and obesity. *Obesity (Silver Spring)*. 2010;18(2):333-339.

6. Reynolds SL, Saito Y, Crimmins EM. The impact of obesity on active life expectancy in older American men and women. *Gerontologist*. 2005;45(4):438-444.

7. Reynolds SL, McIlvane JM. The impact of obesity and arthritis on active life expectancy in older Americans. *Obesity (Silver Spring)*. 2009;17(2):363-369.

8. Whitson HE, Landerman LR, Newman AB, Fried LP, Pieper CF, Cohen HJ. Chronic medical conditions and the sex-based disparity in disability: The cardiovascular health study. *Journals Gerontol - Ser A Biol Sci Med Sci*. 2010;65 A(12):1325-1331.
